# 10-(4-Chloro­phen­yl)-9-(4-fluoro­phen­yl)-3,3,6,6-tetra­methyl-3,4,6,7,9,10-hexa­hydro­acridine-1,8(2*H*,5*H*)-dione

**DOI:** 10.1107/S1600536808025695

**Published:** 2008-08-16

**Authors:** Ling-Ling Zhao, Da Teng

**Affiliations:** a School of Chemistry and Chemical Engineering, Xuzhou Normal University, Xuzhou 221116, People’s Republic of China

## Abstract

The title compound, C_29_H_29_ClFNO_2_, was synthesized by the reaction of 4-fluoro­benzaldehyde, 5,5-dimethyl­cyclo­hexane-1,3-dione and 3-(4-chloro­phenyl­amino)-5,5-dimethyl­cyclo­hex-2-enone in an ionic liquid (1-butyl-3-methyl­imidazolium bromide). X-ray analysis reveals that the 1,4-dihydro­pyridine ring adopts a boat conformation, while each of the attached partially saturated six-membered rings adopts a half-chair conformation. The structure is stabilized by weak C—H⋯O and C—H⋯F hydrogen bonds. The mol­ecule has approximate mirror symmetry; the largest deviation from this symmetry concerns the fluoro- and chloro­phenyl rings.

## Related literature

For related literature, see: Dzierzbicka *et al.* (2001 ▶); Hutchins *et al.* (2003 ▶); Kamal *et al.* (2004 ▶); Li *et al.* (2003 ▶); Petříček *et al.* (2000 ▶); Srivastava & Nizamuddin (2004 ▶); Wang *et al.* (2002 ▶, 2003 ▶).
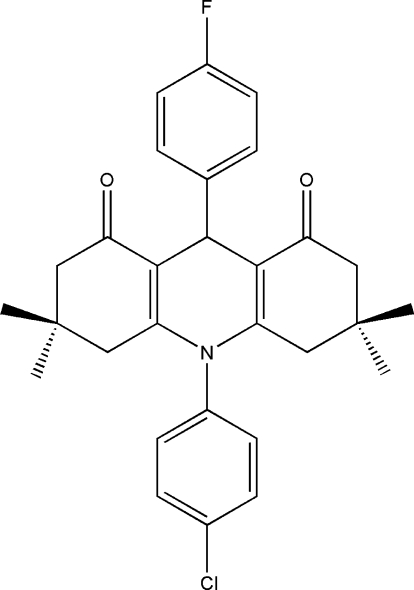


## Experimental

### 

#### Crystal data

C_29_H_29_ClFNO_2_
*M* *_r_* = 477.98
Monoclinic, 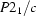

*a* = 12.0985 (12) Å
*b* = 10.9001 (10) Å
*c* = 19.4724 (18) Å
β = 101.231 (3)°
*V* = 2518.7 (4) Å^3^
*Z* = 4
Mo *K*α radiation
μ = 0.18 mm^−1^
*T* = 113 (2) K
0.32 × 0.20 × 0.18 mm

#### Data collection

Rigaku Saturn diffractometer
Absorption correction: multi-scan (*CrystalClear*; Rigaku, 1999 ▶) *T*_min_ = 0.943, *T*_max_ = 0.967
30908 measured reflections
6016 independent reflections
5436 reflections with *I* > 2σ(*I*)
*R*_int_ = 0.040

#### Refinement

*R*[*F* ^2^ > 2σ(*F* ^2^)] = 0.055
*wR*(*F* ^2^) = 0.137
*S* = 1.10
6016 reflections
312 parameters
H-atom parameters constrained
Δρ_max_ = 0.42 e Å^−3^
Δρ_min_ = −0.45 e Å^−3^

### 

Data collection: *CrystalClear* (Rigaku, 1999 ▶); cell refinement: *CrystalClear*; data reduction: *CrystalClear*; program(s) used to solve structure: *SHELXS97* (Sheldrick, 2008 ▶); program(s) used to refine structure: *SHELXL97* (Sheldrick, 2008 ▶); molecular graphics: *SHELXTL* (Sheldrick, 2008 ▶); software used to prepare material for publication: *CrystalStructure* (Rigaku/MSC, 2003 ▶).

## Supplementary Material

Crystal structure: contains datablocks global, I. DOI: 10.1107/S1600536808025695/fb2102sup1.cif

Structure factors: contains datablocks I. DOI: 10.1107/S1600536808025695/fb2102Isup2.hkl

Additional supplementary materials:  crystallographic information; 3D view; checkCIF report

## Figures and Tables

**Table 1:** Hydrogen-bond geometry (Å, °)

*D*—H⋯*A*	*D*—H	H⋯*A*	*D*⋯*A*	*D*—H⋯*A*
C16—H16*C*⋯O2^i^	0.98	2.45	3.359 (2)	153
C11—H11⋯O2^ii^	0.95	2.37	3.286 (2)	160
C10—H10⋯O1^iii^	0.95	2.55	3.474 (2)	165
C16—H16*A*⋯O1^iv^	0.98	2.59	3.528 (2)	159
C17—H17*A*⋯F1^v^	0.98	2.45	3.373 (2)	157

Symmetry codes: (i) 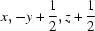; (ii) 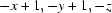; (iii) 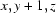; (iv) 
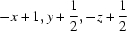
; (v) 
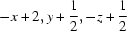
.

**Fig. 1.**
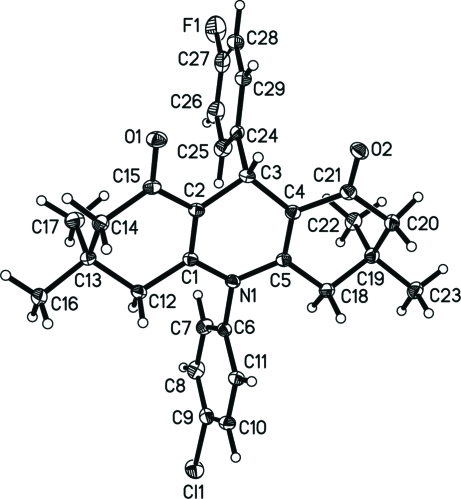
The title molecule with the displacement ellipsoids shown at the 50% probability level.

**Fig. 2.**
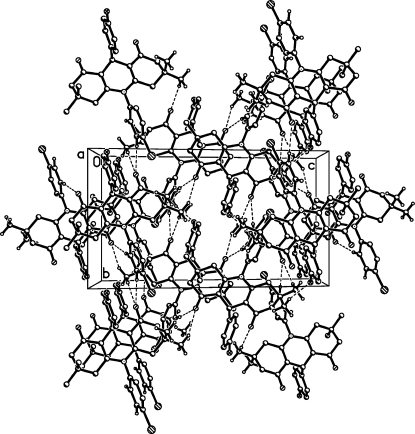
The molecular packing including the hydrogen-bonding network.

## supplementary crystallographic information

### Comment

Acridine derivatives are well-known compounds because of their pharmacological profile as anticancer agents (Hutchins *et al.*, 2003) and potential DNA-binding agents (Kamal *et al.*, 2004). Acridine derivatives have also been reported to possess antitumor activity (Dzierzbicka *et al.*, 2001) as well as fungicidal activity (Srivastava & Nizamuddin, 2004). Here we report the crystal structure of 10-(4-chlorophenyl)-9-(4-fluorophenyl)-3,4,6,7-tetrahydro-3,3,6,6- tetramethylacridine-1,8(2*H*,5*H*,9*H*,10*H*)-dione.

The X-ray crystal structure determination indicates that the central 1,4-dihydropyridine ring C1/C2···N1 is slightly distorted and adopts the boat conformation. The atoms C1, C2, C4 and C5 are coplanar, with C3 and N1 deviating from the plane by 0.286 (3) and 0.109 (2) Å, respectively. The similar distortions have been observed in the structures of 3,3,6,6-tetramethyl-9-(4-chlorophenyl)-10-(4-methylphenyl)- 1,2,3,4,5,6,7,8,9,10-decahydroacridine-1,8-dione [0.192 (3) and 0.091 (3) Å for C13 and N, respectively; Wang *et al.*, 2003) and 3,3,6,6-tetramethyl-9-(3,4-methylenedioxylphenyl)-1,2,3,4,5,6,7,8,9,10- decahydroacridine-1,8-dione [0.313 (7) and 0.107 (7) Å for C7 and N1, respectively; Li *et al.*, 2003).

The two outer six-membered rings of the acridine group adopt half-chair conformations; the atoms C13 and C19 deviate from the mean planes defined by C1, C2, C14, C15, C12 and C4, C5, C18, C20, C21 by 0.657 (2) and 0.668 (2) Å, respectively. A similar conformation has been found in the structure of 7,7-dimethyl-2-amino-3-cyano-4-(3,4-methylenedioxylphenyl)-5-oxo-5,6,7,8- tetrahydro-4*H*-benzo-[*b*]-pyran (Wang *et al.*, 2002).

The 4-fluorophenyl ring is nearly perpendicular to the plane defined by the atoms C1—C2—C4—C5, forming a dihedral angle of 87.9 (1)°. The molecule has an approximate mirror symmetry. For example, the atoms C14 and C20 of the acridine groups are deviated by about 0.25Å. (The calculation has been carried out with the help of JANA2000 (Petříček *et al.*, 2000). The largest deviation from this symmetry concerns the fluoro- and the chlorophenyl rings whose planes contain 9.3 (1)°.

The weak hydrogen bonds of C—H···O and C—H···F are listed in Table 1. The weak intermolecular hydrogen bonds of C—H···O and C—H···F link the molecules (Fig. 2).

### Experimental

The title compound was prepared by the reaction of 4-fluorobenzaldehyde (2 mmol, 0.248 g), 5,5-dimethyl-1,3-cyclohexanedione (2 mmol, 0.280 g) and 3-(4-chlorophenylamino)-5,5-dimethylcyclohex-2-enone (2 mmol, 0.498 g) in the ionic liquid of [Bmim]Br (1-butyl-3-methylimidazolium bromide) (10 ml) at 353 K. After the reaction had completed (monitored by TLC, about 6 h), the reactants were cooled to room temperature. The generated yellow solid was filtered off, and washed with small amount of water. The block crystals (about 0.2 mm in length, width and height respectively) suitable for X-ray diffraction were obtained by slow evaporation from ethanol solution. M.p. 583–585 K.

### Refinement

In the structure all the H atoms were discernible in the difference electron density map. However, they were constrained by the riding model approximation. C—H_methyl_ = 0.98 Å; C—H_methylene_ = 0.99 Å; C—H_methine_ = 1.00 Å; C—H_aryl_ = 0.95 Å; *U*_iso_H_methyl_ = 1.5*U*_eq_(C_methyl_); *U*_iso_H_aryl_ = 1.2*U*_eq_(C_aryl_, C_methylene_ and C_methine_).

### Crystal data

**Table:**

C_29_H_29_ClFNO_2_	*F*_000_ = 1008
*M**_r_* = 477.98	*D*_x_ = 1.260 Mg m^−^^3^
Monoclinic, *P*2_1_/*c*	Melting point = 583–585 K
Hall symbol: -P 2ybc	Mo *K*α radiation λ = 0.71070 Å
*a* = 12.0985 (12) Å	Cell parameters from 8097 reflections
*b* = 10.9001 (10) Å	θ = 1.7–27.9º
*c* = 19.4724 (18) Å	µ = 0.19 mm^−^^1^
β = 101.231 (3)º	*T* = 113 (2) K
*V* = 2518.7 (4) Å^3^	Block, yellow
*Z* = 4	0.32 × 0.20 × 0.18 mm

### Data collection

**Table:**

Rigaku Saturn diffractometer	6016 independent reflections
Radiation source: rotating anode	5436 reflections with *I* > 2σ(*I*)
Monochromator: confocal	*R*_int_ = 0.040
Detector resolution: 14.63 pixels mm^-1^	θ_max_ = 27.9º
*T* = 113(2) K	θ_min_ = 1.7º
ω scans	*h* = −15→15
Absorption correction: multi-scan(CrystalClear; Rigaku, 1999)	*k* = −14→14
*T*_min_ = 0.943, *T*_max_ = 0.967	*l* = −25→25
30908 measured reflections	

### Refinement

**Table:**

Refinement on *F*^2^	Secondary atom site location: difference Fourier map
Least-squares matrix: full	Hydrogen site location: difference Fourier map
*R*[*F*^2^ > 2σ(*F*^2^)] = 0.055	H-atom parameters constrained
*wR*(*F*^2^) = 0.137	*w* = 1/[σ^2^(*F*_o_^2^) + (0.0579*P*)^2^ + 1.0431*P*] where *P* = (*F*_o_^2^ + 2*F*_c_^2^)/3
*S* = 1.10	(Δ/σ)_max_ = 0.001
6016 reflections	Δρ_max_ = 0.42 e Å^−^^3^
312 parameters	Δρ_min_ = −0.45 e Å^−^^3^
112 constraints	Extinction correction: SHELXL97 (Sheldrick, 2008), Fc^*^=kFc[1+0.001xFc^2^λ^3^/sin(2θ)]^-1/4^
Primary atom site location: structure-invariant direct methods	Extinction coefficient: 0.0058 (9)

### Special details

**Table:**

Geometry. All esds (except the esd in the dihedral angle between two l.s. planes) are estimated using the full covariance matrix. The cell esds are taken into account individually in the estimation of esds in distances, angles and torsion angles; correlations between esds in cell parameters are only used when they are defined by crystal symmetry. An approximate (isotropic) treatment of cell esds is used for estimating esds involving l.s. planes.
Refinement. Refinement of *F*^2^ against ALL reflections. The weighted *R*-factor *wR* and goodness of fit *S* are based on *F*^2^, conventional *R*-factors *R* are based on *F*, with *F* set to zero for negative *F*^2^. The threshold expression of *F*^2^ > σ(*F*^2^) is used only for calculating *R*-factors(gt) *etc*. and is not relevant to the choice of reflections for refinement. *R*-factors based on *F*^2^ are statistically about twice as large as those based on *F*, and *R*- factors based on ALL data will be even larger.

### Fractional atomic coordinates and isotropic or equivalent isotropic displacement parameters (Å^2^)

**Table:**

	*x*	*y*	*z*	*U*_iso_*/*U*_eq_	
Cl1	0.86649 (5)	1.04288 (4)	0.25315 (3)	0.04213 (16)	
F1	1.03322 (14)	0.02721 (16)	0.07820 (8)	0.0761 (5)	
O1	0.56729 (11)	0.16766 (11)	0.17692 (7)	0.0326 (3)	
O2	0.57270 (11)	0.31474 (12)	−0.06509 (7)	0.0330 (3)	
N1	0.70646 (12)	0.56013 (12)	0.13279 (7)	0.0229 (3)	
C1	0.67413 (13)	0.47215 (14)	0.17771 (8)	0.0219 (3)	
C2	0.64094 (13)	0.35886 (15)	0.15353 (8)	0.0223 (3)	
C3	0.64825 (14)	0.31701 (14)	0.08070 (9)	0.0231 (3)	
H3	0.5793	0.2681	0.0614	0.028*	
C4	0.65130 (13)	0.42790 (15)	0.03444 (9)	0.0231 (3)	
C5	0.68642 (13)	0.53925 (14)	0.06063 (9)	0.0224 (3)	
C6	0.74535 (14)	0.67913 (14)	0.16009 (8)	0.0228 (3)	
C7	0.85853 (15)	0.69498 (17)	0.18908 (10)	0.0304 (4)	
H7	0.9099	0.6289	0.1895	0.036*	
C8	0.89647 (16)	0.80758 (18)	0.21746 (10)	0.0336 (4)	
H8	0.9738	0.8194	0.2376	0.040*	
C9	0.82031 (15)	0.90184 (16)	0.21587 (9)	0.0278 (4)	
C10	0.70815 (15)	0.88861 (15)	0.18595 (9)	0.0269 (4)	
H10	0.6576	0.9557	0.1844	0.032*	
C11	0.67004 (14)	0.77543 (15)	0.15803 (9)	0.0244 (3)	
H11	0.5927	0.7642	0.1376	0.029*	
C12	0.67473 (15)	0.51183 (15)	0.25200 (9)	0.0263 (4)	
H12A	0.6074	0.5631	0.2527	0.032*	
H12B	0.7422	0.5631	0.2686	0.032*	
C13	0.67515 (15)	0.40361 (15)	0.30251 (9)	0.0264 (4)	
C14	0.58278 (15)	0.31382 (16)	0.26947 (9)	0.0274 (4)	
H14A	0.5842	0.2417	0.3006	0.033*	
H14B	0.5085	0.3541	0.2656	0.033*	
C15	0.59618 (13)	0.27108 (15)	0.19831 (9)	0.0242 (3)	
C16	0.65091 (19)	0.45153 (17)	0.37181 (10)	0.0365 (4)	
H16A	0.5787	0.4952	0.3632	0.055*	
H16B	0.7111	0.5077	0.3931	0.055*	
H16C	0.6474	0.3825	0.4035	0.055*	
C17	0.78922 (16)	0.33872 (18)	0.31572 (11)	0.0367 (4)	
H17A	0.8487	0.3977	0.3342	0.055*	
H17B	0.8031	0.3043	0.2717	0.055*	
H17C	0.7890	0.2725	0.3497	0.055*	
C18	0.70364 (15)	0.64547 (15)	0.01412 (9)	0.0263 (4)	
H18A	0.7669	0.6965	0.0386	0.032*	
H18B	0.6350	0.6970	0.0056	0.032*	
C19	0.72913 (15)	0.60365 (16)	−0.05647 (9)	0.0276 (4)	
C20	0.63778 (15)	0.51338 (17)	−0.08888 (9)	0.0293 (4)	
H20A	0.5663	0.5589	−0.1036	0.035*	
H20B	0.6584	0.4777	−0.1314	0.035*	
C21	0.61825 (14)	0.41026 (16)	−0.04108 (9)	0.0264 (4)	
C22	0.84573 (16)	0.54320 (18)	−0.04596 (10)	0.0338 (4)	
H22A	0.8465	0.4697	−0.0168	0.051*	
H22B	0.9029	0.6011	−0.0228	0.051*	
H22C	0.8622	0.5201	−0.0916	0.051*	
C23	0.72650 (17)	0.71613 (17)	−0.10400 (10)	0.0347 (4)	
H23A	0.7434	0.6909	−0.1491	0.052*	
H23B	0.7829	0.7758	−0.0818	0.052*	
H23C	0.6515	0.7537	−0.1114	0.052*	
C24	0.75157 (15)	0.23635 (15)	0.08100 (9)	0.0262 (4)	
C25	0.85885 (16)	0.28331 (19)	0.10536 (10)	0.0350 (4)	
H25	0.8671	0.3649	0.1227	0.042*	
C26	0.95412 (18)	0.2128 (2)	0.10467 (11)	0.0443 (5)	
H26	1.0274	0.2449	0.1214	0.053*	
C27	0.9395 (2)	0.0961 (2)	0.07926 (11)	0.0480 (6)	
C28	0.8360 (2)	0.0456 (2)	0.05529 (11)	0.0479 (6)	
H28	0.8289	−0.0363	0.0382	0.057*	
C29	0.74091 (18)	0.11699 (17)	0.05649 (10)	0.0357 (4)	
H29	0.6680	0.0834	0.0403	0.043*	

### Atomic displacement parameters (Å^2^)

**Table:**

	*U*^11^	*U*^22^	*U*^33^	*U*^12^	*U*^13^	*U*^23^
Cl1	0.0539 (3)	0.0306 (3)	0.0401 (3)	−0.0193 (2)	0.0050 (2)	−0.00797 (19)
F1	0.0758 (10)	0.1001 (13)	0.0550 (9)	0.0666 (10)	0.0195 (8)	0.0108 (8)
O1	0.0356 (7)	0.0234 (6)	0.0404 (7)	−0.0077 (5)	0.0110 (6)	−0.0044 (5)
O2	0.0345 (7)	0.0322 (7)	0.0303 (7)	−0.0003 (5)	0.0012 (5)	−0.0087 (5)
N1	0.0274 (7)	0.0189 (6)	0.0234 (7)	−0.0022 (5)	0.0071 (5)	−0.0024 (5)
C1	0.0220 (8)	0.0207 (8)	0.0236 (8)	0.0005 (6)	0.0060 (6)	−0.0010 (6)
C2	0.0210 (8)	0.0212 (8)	0.0255 (8)	0.0014 (6)	0.0062 (6)	−0.0019 (6)
C3	0.0240 (8)	0.0199 (8)	0.0255 (8)	−0.0012 (6)	0.0051 (6)	−0.0040 (6)
C4	0.0214 (8)	0.0228 (8)	0.0249 (8)	0.0013 (6)	0.0042 (6)	−0.0019 (6)
C5	0.0215 (8)	0.0215 (8)	0.0248 (8)	0.0021 (6)	0.0058 (6)	−0.0007 (6)
C6	0.0272 (8)	0.0195 (8)	0.0225 (8)	−0.0033 (6)	0.0069 (6)	−0.0011 (6)
C7	0.0247 (9)	0.0297 (9)	0.0361 (10)	0.0017 (7)	0.0042 (7)	0.0027 (7)
C8	0.0251 (9)	0.0359 (10)	0.0374 (10)	−0.0087 (7)	0.0000 (7)	0.0017 (8)
C9	0.0338 (9)	0.0255 (8)	0.0242 (8)	−0.0101 (7)	0.0058 (7)	−0.0005 (7)
C10	0.0297 (9)	0.0215 (8)	0.0310 (9)	−0.0012 (7)	0.0093 (7)	−0.0027 (7)
C11	0.0231 (8)	0.0227 (8)	0.0281 (8)	−0.0023 (6)	0.0065 (6)	−0.0025 (6)
C12	0.0355 (9)	0.0201 (8)	0.0248 (8)	−0.0015 (7)	0.0097 (7)	−0.0032 (6)
C13	0.0335 (9)	0.0199 (8)	0.0253 (8)	−0.0007 (7)	0.0046 (7)	−0.0001 (6)
C14	0.0311 (9)	0.0244 (8)	0.0282 (9)	−0.0023 (7)	0.0091 (7)	0.0014 (7)
C15	0.0203 (8)	0.0207 (8)	0.0314 (9)	0.0002 (6)	0.0044 (6)	−0.0022 (6)
C16	0.0572 (13)	0.0263 (9)	0.0267 (9)	−0.0019 (8)	0.0100 (8)	0.0006 (7)
C17	0.0349 (10)	0.0314 (10)	0.0394 (11)	0.0016 (8)	−0.0038 (8)	−0.0039 (8)
C18	0.0303 (9)	0.0230 (8)	0.0268 (9)	0.0019 (7)	0.0084 (7)	0.0003 (7)
C19	0.0307 (9)	0.0282 (9)	0.0249 (8)	0.0044 (7)	0.0081 (7)	0.0028 (7)
C20	0.0307 (9)	0.0318 (9)	0.0243 (8)	0.0053 (7)	0.0028 (7)	0.0005 (7)
C21	0.0217 (8)	0.0286 (9)	0.0283 (9)	0.0059 (7)	0.0039 (6)	−0.0034 (7)
C22	0.0292 (9)	0.0388 (10)	0.0356 (10)	0.0058 (8)	0.0121 (8)	0.0053 (8)
C23	0.0415 (11)	0.0330 (10)	0.0309 (10)	0.0053 (8)	0.0106 (8)	0.0067 (8)
C24	0.0332 (9)	0.0248 (8)	0.0221 (8)	0.0069 (7)	0.0096 (7)	0.0026 (6)
C25	0.0299 (10)	0.0371 (10)	0.0395 (11)	0.0063 (8)	0.0107 (8)	0.0043 (8)
C26	0.0334 (11)	0.0602 (14)	0.0423 (12)	0.0169 (10)	0.0146 (9)	0.0142 (10)
C27	0.0546 (14)	0.0618 (15)	0.0310 (10)	0.0382 (12)	0.0168 (9)	0.0116 (10)
C28	0.0770 (17)	0.0372 (11)	0.0303 (11)	0.0299 (11)	0.0124 (10)	−0.0012 (8)
C29	0.0515 (12)	0.0278 (9)	0.0273 (9)	0.0104 (8)	0.0058 (8)	−0.0018 (7)

### Geometric parameters (Å, °)

**Table:**

Cl1—C9	1.7449 (18)	C14—H14A	0.9900
F1—C27	1.364 (2)	C14—H14B	0.9900
O1—C15	1.229 (2)	C16—H16A	0.9800
O2—C21	1.227 (2)	C16—H16B	0.9800
N1—C5	1.397 (2)	C16—H16C	0.9800
N1—C1	1.404 (2)	C17—H17A	0.9800
N1—C6	1.445 (2)	C17—H17B	0.9800
C1—C2	1.354 (2)	C17—H17C	0.9800
C1—C12	1.509 (2)	C18—C19	1.535 (2)
C2—C15	1.467 (2)	C18—H18A	0.9900
C2—C3	1.509 (2)	C18—H18B	0.9900
C3—C4	1.512 (2)	C19—C20	1.522 (3)
C3—C24	1.527 (2)	C19—C23	1.533 (2)
C3—H3	1.0000	C19—C22	1.534 (2)
C4—C5	1.353 (2)	C20—C21	1.507 (3)
C4—C21	1.460 (2)	C20—H20A	0.9900
C5—C18	1.509 (2)	C20—H20B	0.9900
C6—C11	1.385 (2)	C22—H22A	0.9800
C6—C7	1.387 (2)	C22—H22B	0.9800
C7—C8	1.387 (3)	C22—H22C	0.9800
C7—H7	0.9500	C23—H23A	0.9800
C8—C9	1.376 (3)	C23—H23B	0.9800
C8—H8	0.9500	C23—H23C	0.9800
C9—C10	1.376 (2)	C24—C29	1.383 (2)
C10—C11	1.390 (2)	C24—C25	1.390 (3)
C10—H10	0.9500	C25—C26	1.388 (3)
C11—H11	0.9500	C25—H25	0.9500
C12—C13	1.535 (2)	C26—C27	1.364 (3)
C12—H12A	0.9900	C26—H26	0.9500
C12—H12B	0.9900	C27—C28	1.364 (4)
C13—C17	1.527 (2)	C28—C29	1.393 (3)
C13—C16	1.528 (2)	C28—H28	0.9500
C13—C14	1.530 (2)	C29—H29	0.9500
C14—C15	1.500 (2)		
			
C5—N1—C1	120.03 (13)	H16A—C16—H16B	109.5
C5—N1—C6	119.75 (13)	C13—C16—H16C	109.5
C1—N1—C6	119.62 (13)	H16A—C16—H16C	109.5
C2—C1—N1	120.39 (15)	H16B—C16—H16C	109.5
C2—C1—C12	122.73 (15)	C13—C17—H17A	109.5
N1—C1—C12	116.85 (13)	C13—C17—H17B	109.5
C1—C2—C15	120.44 (15)	H17A—C17—H17B	109.5
C1—C2—C3	122.33 (15)	C13—C17—H17C	109.5
C15—C2—C3	117.23 (14)	H17A—C17—H17C	109.5
C2—C3—C4	109.33 (13)	H17B—C17—H17C	109.5
C2—C3—C24	111.60 (13)	C5—C18—C19	112.63 (14)
C4—C3—C24	110.34 (13)	C5—C18—H18A	109.1
C2—C3—H3	108.5	C19—C18—H18A	109.1
C4—C3—H3	108.5	C5—C18—H18B	109.1
C24—C3—H3	108.5	C19—C18—H18B	109.1
C5—C4—C21	120.15 (15)	H18A—C18—H18B	107.8
C5—C4—C3	122.35 (15)	C20—C19—C23	109.80 (15)
C21—C4—C3	117.47 (14)	C20—C19—C22	110.63 (15)
C4—C5—N1	120.36 (15)	C23—C19—C22	109.55 (15)
C4—C5—C18	122.15 (15)	C20—C19—C18	107.87 (14)
N1—C5—C18	117.48 (14)	C23—C19—C18	108.60 (14)
C11—C6—C7	120.61 (15)	C22—C19—C18	110.36 (14)
C11—C6—N1	120.28 (14)	C21—C20—C19	114.50 (14)
C7—C6—N1	119.11 (15)	C21—C20—H20A	108.6
C6—C7—C8	119.77 (17)	C19—C20—H20A	108.6
C6—C7—H7	120.1	C21—C20—H20B	108.6
C8—C7—H7	120.1	C19—C20—H20B	108.6
C9—C8—C7	118.91 (16)	H20A—C20—H20B	107.6
C9—C8—H8	120.5	O2—C21—C4	120.67 (16)
C7—C8—H8	120.5	O2—C21—C20	120.69 (16)
C10—C9—C8	122.13 (16)	C4—C21—C20	118.61 (15)
C10—C9—Cl1	118.55 (14)	C19—C22—H22A	109.5
C8—C9—Cl1	119.32 (14)	C19—C22—H22B	109.5
C9—C10—C11	118.90 (16)	H22A—C22—H22B	109.5
C9—C10—H10	120.6	C19—C22—H22C	109.5
C11—C10—H10	120.6	H22A—C22—H22C	109.5
C6—C11—C10	119.66 (15)	H22B—C22—H22C	109.5
C6—C11—H11	120.2	C19—C23—H23A	109.5
C10—C11—H11	120.2	C19—C23—H23B	109.5
C1—C12—C13	113.13 (13)	H23A—C23—H23B	109.5
C1—C12—H12A	109.0	C19—C23—H23C	109.5
C13—C12—H12A	109.0	H23A—C23—H23C	109.5
C1—C12—H12B	109.0	H23B—C23—H23C	109.5
C13—C12—H12B	109.0	C29—C24—C25	118.83 (17)
H12A—C12—H12B	107.8	C29—C24—C3	121.26 (16)
C17—C13—C16	109.41 (15)	C25—C24—C3	119.90 (15)
C17—C13—C14	109.67 (14)	C26—C25—C24	121.0 (2)
C16—C13—C14	109.88 (15)	C26—C25—H25	119.5
C17—C13—C12	110.72 (15)	C24—C25—H25	119.5
C16—C13—C12	109.02 (14)	C27—C26—C25	118.1 (2)
C14—C13—C12	108.12 (14)	C27—C26—H26	121.0
C15—C14—C13	112.68 (14)	C25—C26—H26	121.0
C15—C14—H14A	109.1	F1—C27—C26	118.0 (2)
C13—C14—H14A	109.1	F1—C27—C28	118.9 (2)
C15—C14—H14B	109.1	C26—C27—C28	123.11 (19)
C13—C14—H14B	109.1	C27—C28—C29	118.3 (2)
H14A—C14—H14B	107.8	C27—C28—H28	120.8
O1—C15—C2	120.69 (16)	C29—C28—H28	120.8
O1—C15—C14	121.55 (15)	C24—C29—C28	120.6 (2)
C2—C15—C14	117.72 (14)	C24—C29—H29	119.7
C13—C16—H16A	109.5	C28—C29—H29	119.7
C13—C16—H16B	109.5		
			
C5—N1—C1—C2	11.0 (2)	C1—C12—C13—C17	71.36 (18)
C6—N1—C1—C2	−177.87 (14)	C1—C12—C13—C16	−168.23 (15)
C5—N1—C1—C12	−166.96 (14)	C1—C12—C13—C14	−48.81 (19)
C6—N1—C1—C12	4.1 (2)	C17—C13—C14—C15	−64.45 (19)
N1—C1—C2—C15	−173.71 (14)	C16—C13—C14—C15	175.25 (14)
C12—C1—C2—C15	4.2 (2)	C12—C13—C14—C15	56.38 (19)
N1—C1—C2—C3	6.5 (2)	C1—C2—C15—O1	−179.08 (15)
C12—C1—C2—C3	−175.64 (15)	C3—C2—C15—O1	0.7 (2)
C1—C2—C3—C4	−22.1 (2)	C1—C2—C15—C14	3.3 (2)
C15—C2—C3—C4	158.08 (14)	C3—C2—C15—C14	−176.84 (14)
C1—C2—C3—C24	100.24 (18)	C13—C14—C15—O1	147.61 (16)
C15—C2—C3—C24	−79.58 (18)	C13—C14—C15—C2	−34.8 (2)
C2—C3—C4—C5	23.5 (2)	C4—C5—C18—C19	−25.5 (2)
C24—C3—C4—C5	−99.62 (18)	N1—C5—C18—C19	155.53 (14)
C2—C3—C4—C21	−158.60 (14)	C5—C18—C19—C20	52.13 (18)
C24—C3—C4—C21	78.30 (18)	C5—C18—C19—C23	171.08 (15)
C21—C4—C5—N1	173.05 (14)	C5—C18—C19—C22	−68.83 (19)
C3—C4—C5—N1	−9.1 (2)	C23—C19—C20—C21	−169.46 (14)
C21—C4—C5—C18	−5.9 (2)	C22—C19—C20—C21	69.51 (19)
C3—C4—C5—C18	171.93 (15)	C18—C19—C20—C21	−51.28 (19)
C1—N1—C5—C4	−9.7 (2)	C5—C4—C21—O2	−170.52 (16)
C6—N1—C5—C4	179.21 (15)	C3—C4—C21—O2	11.5 (2)
C1—N1—C5—C18	169.32 (14)	C5—C4—C21—C20	7.5 (2)
C6—N1—C5—C18	−1.8 (2)	C3—C4—C21—C20	−170.44 (14)
C5—N1—C6—C11	79.2 (2)	C19—C20—C21—O2	−159.05 (16)
C1—N1—C6—C11	−91.90 (19)	C19—C20—C21—C4	22.9 (2)
C5—N1—C6—C7	−101.61 (19)	C2—C3—C24—C29	120.14 (17)
C1—N1—C6—C7	87.3 (2)	C4—C3—C24—C29	−118.09 (17)
C11—C6—C7—C8	1.2 (3)	C2—C3—C24—C25	−60.7 (2)
N1—C6—C7—C8	−177.99 (16)	C4—C3—C24—C25	61.0 (2)
C6—C7—C8—C9	−0.3 (3)	C29—C24—C25—C26	0.6 (3)
C7—C8—C9—C10	−1.2 (3)	C3—C24—C25—C26	−178.51 (17)
C7—C8—C9—Cl1	178.58 (14)	C24—C25—C26—C27	0.1 (3)
C8—C9—C10—C11	1.7 (3)	C25—C26—C27—F1	179.58 (18)
Cl1—C9—C10—C11	−178.07 (13)	C25—C26—C27—C28	−0.7 (3)
C7—C6—C11—C10	−0.7 (3)	F1—C27—C28—C29	−179.85 (18)
N1—C6—C11—C10	178.50 (15)	C26—C27—C28—C29	0.4 (3)
C9—C10—C11—C6	−0.7 (3)	C25—C24—C29—C28	−0.9 (3)
C2—C1—C12—C13	20.1 (2)	C3—C24—C29—C28	178.23 (16)
N1—C1—C12—C13	−161.93 (14)	C27—C28—C29—C24	0.4 (3)

### Hydrogen-bond geometry (Å, °)

**Table:**

*D*—H···*A*	*D*—H	H···*A*	*D*···*A*	*D*—H···*A*
C16—H16C···O2^i^	0.98	2.45	3.359 (2)	153
C11—H11···O2^ii^	0.95	2.37	3.286 (2)	160
C10—H10···O1^iii^	0.95	2.55	3.474 (2)	165
C16—H16A···O1^iv^	0.98	2.59	3.528 (2)	159
C17—H17A···F1^v^	0.98	2.45	3.373 (2)	157

Symmetry codes: (i) *x*, −*y*+1/2, *z*+1/2; (ii) −*x*+1, −*y*+1, −*z*; (iii) *x*, *y*+1, *z*; (iv) −*x*+1, *y*+1/2, −*z*+1/2; (v) −*x*+2, *y*+1/2, −*z*+1/2.
